# Clinical outcomes of atrial fibrillation screening: a meta-analysis of randomized controlled trials

**DOI:** 10.1080/07853890.2025.2457522

**Published:** 2025-01-25

**Authors:** Ville Langén, Aleksi K. Winstén, K. E. Juhani Airaksinen, Konsta Teppo

**Affiliations:** a Division of Medicine, Turku University Hospital and University of Turku, Turku, Finland; b Faculty of Medicine, Department of Mathematics and Statistics, University of Turku, Turku, Finland; c Turku University Hospital and University of Turku, Turku, Finland; d Heart Centre, Turku University Hospital, Turku, Finland; e Biotechnology Unit, Department of Life Technologies, University of Turku, Turku, Finland

**Keywords:** Atrial fibrillation, screening, outcomes, stroke, bleeding, mortality

## Abstract

**Background:**

Several randomized controlled trials (RCTs) have investigated the benefits of atrial fibrillation (AF) screening. However, since none have shown a significant reduction in stroke rates, the impact of screening on clinical outcomes remains uncertain.

**Materials and methods:**

We conducted a systematic review and meta-analysis of RCTs reporting clinical outcomes of systematic AF screening in participants without known AF. Pooled risk ratios (RRs) were computed for all-cause stroke or systemic embolism, major bleeding, and all-cause mortality, comparing screening with no screening.

**Results:**

Seven RCTs encompassing 76 458 participants were identified. One trial utilized implantable loop recorders for rhythm monitoring, while the others employed non-invasive screening methods. Pooled results indicated that AF screening was associated with a significant reduction in all-cause stroke or systemic embolism (RR 0.932, 95% CI 0.873–0.996, I^2^ = 0%, *p* = 0.037), but had no effect on major bleeding (RR 0.996, 95% CI 0.935–1.060, I^2^ = 0%, *p* = 0.876) or all-cause mortality (RR 0.987, 95% CI 0.945–1.031, I^2^ = 0%, *p* = 0.550). We estimated a number needed to screen of 148 to prevent one stroke or systemic embolism over a 10-year period in a population of 75-year-olds. When only non-invasive screening methods were considered, the reduction in strokes was not statistically significant (RR 0.942, 95% CI 0.880–1.008, I^2^ = 0%, *p* = 0.083).

**Conclusions:**

Systematic AF screening is associated with a modest yet statistically significant 7% relative reduction in stroke and systemic embolism, with no observed impact on major bleeding or all-cause mortality.

## Introduction

Atrial fibrillation (AF) is the most common sustained arrhythmia and a major risk factor for ischemic stroke [1]. Fortunately, once AF is diagnosed, the stroke risk related to it can be effectively reduced by oral anticoagulation (OAC) [2]. However, AF episodes are often short and asymptomatic, and therefore, AF can remain undiagnosed—and untreated. In fact, ischemic stroke is estimated to be the first manifestation of AF in approximately one in four patients [3,4].

To reduce the burden of ischemic stroke, screening for asymptomatic patients has been hypothesized to identify those with subclinical, undiagnosed AF, in whom initiating OAC therapy could prevent future strokes. Several randomized trials, employing various screening methods and outcome measures, have investigated this hypothesis over the years, yielding mixed findings. Most trials have focused on individuals over 65 years with stroke risk factors, as AF is more prevalent in this population and associated with a higher risk of stroke [5]. Screening has generally led to an increased detection of AF and, consequently, a higher use of OACs; however, this has translated into only minimal or nonexistent benefits in stroke prevention. A previous meta-analysis suggested that AF screening may be associated with a small reduction in stroke (relative risk 0.91, 95% CI 0.84–0.99), but this finding was not replicated in another meta-analysis that concentrated only on non-invasive AF screening tools [6,7]. Importantly, after these meta-analyses, two randomized studies on AF screening, the GUARD-AF and STROKESTOP II trials, have been published. They have provided new robust data on the outcome benefits of AF screening, with their findings being neutral regarding stroke prevention [8,9].

Notwithstanding the disparities in the results of the AF screening trials, the recent European guidelines on AF management give a relatively strong IIa level recommendation to consider population-based screening for AF with a non-invasive approach in individuals over the age of 75 years or those over 65 years with other stroke risk factors [10]. To inform policy-making regarding the overall benefits of AF screening, we undertook a focused review to summarize existing trial-based evidence on the outcome benefits of systematic screening for AF. We also aimed to provide updated pooled estimates of its impact on stroke or systemic embolism, major bleeding, and all-cause mortality.

## Methods

### Data selection

We conducted a systematic literature review by searching the PubMed database for randomized controlled trials on October 14, 2024 with the following search terms: ‘atrial fibrillation’ AND ‘screening’ AND (‘stroke’ OR ‘bleeding’ OR ‘mortality’ OR ‘outcomes’). We included only randomized trials that enrolled participants without known AF, screened for AF using electrocardiogram-based methods, and reported stroke, major bleeding, or all-cause mortality outcomes between individuals randomized to be invited for screening and the controls. Only studies published in English were included. We focused on systematic screening for AF and the primary prevention of stroke, thus excluding studies that assessed only opportunistic screening or that focused on post-stroke patients. The current meta-analysis was performed in accordance with the Preferred Reporting Items for Systematic Reviews and Network Meta-Analyses (PRISMA) guidelines. A protocol for this meta-analysis was prospectively registered at International prospective register of systematic reviews (PROSPERO ID 2024 CRD42024601424). Two independent investigators (KT and VL) extracted the study features, baseline characteristics, screening strategies and clinical outcomes.

### Outcomes

This meta-analysis focused on all-cause stroke or systemic embolism, major bleeding, and all-cause mortality. To address heterogeneity in the reported trial outcome measures, we accepted composite outcomes with ischemic strokes, hemorrhagic strokes, and transient ischemic attacks in the analysis of stroke or systemic embolism.

### Statistical analyses

A pairwise meta-analysis was conducted to compare AF screening with no screening, using an intention-to-treat approach. Data were pooled using random-effects models with Restricted Maximum Likelihood Estimation, and summary estimates were reported as risk ratios (RR) with 95% confidence intervals (CI). A random-effects model was chosen to account for variability in study designs and treatment effects. If the pooled RR for stroke reached statistical significance, absolute risk reduction and a number needed to screen with 95% CIs were estimated. This analysis assumed a 10% baseline stroke risk over ten years, based on observations from a real-life population of 75-year-olds [11,12]. Moreover, we conducted separate analyses for studies that exclusively used non-invasive screening methods, as well as for those with clinical endpoints as the primary outcome measures. Statistical heterogeneity was assessed using the I^2^ statistic, with a pre-specified threshold of 50% to indicate significant heterogeneity [13]. Publication bias was not assessed, as fewer than ten studies were included [14]. Statistical significance was determined based on 95% CIs of the RR estimates. All analyses were performed with R (version 4.2.2, R Core Team, Vienna, Austria) using the ‘meta’ package (version 7.0.0). In the interest of research reproducibility, we have deposited the codes used in these analyses in the Zenodo repository (DOI:10.5281/zenodo.13984037).

## Results

**Figure 1.**
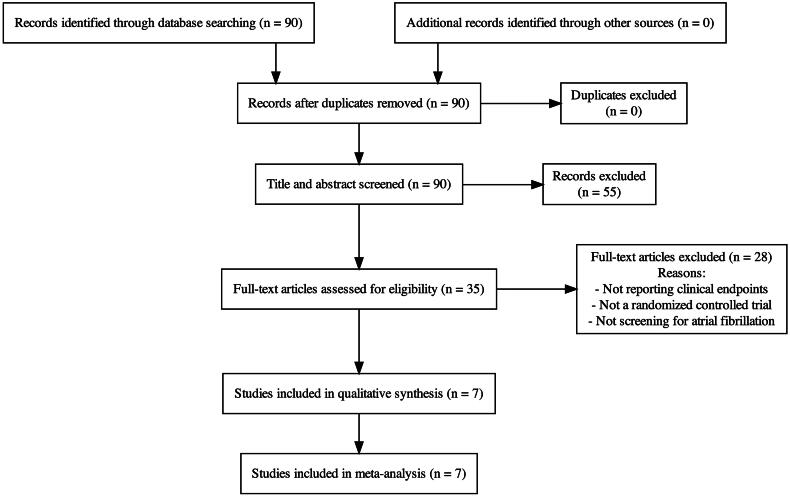
Study selection diagram.

We included seven trials with a total of 76 458 participants that reported outcomes related to stroke or systemic embolism, major bleeding, or all-cause mortality (Figure 1). All studies included older patients with stroke risk factors, with a weighted mean age of 75.7 years and a follow-up duration of 5.1 years. Four included trials had clinical outcomes as their primary endpoints and were initially powered to detect outcome differences; however, enrollment in the GUARD-AF trial was prematurely terminated due to the COVID-19 pandemic. The LOOP trial used implantable loop recorder to monitor heart rhythm, whereas all other identified studies applied non-invasive methods (Table 1). The STROKESTOP was the only trial to report a statistically significant primary outcome—a composite of ischemic or hemorrhagic stroke, systemic embolism, hospitalization for bleeding, or death from any cause—while all other trials showed neutral results regarding clinical outcomes. The two STROKESTOP trials had the highest number of outcome events, giving them the greatest weight when calculating the pooled risk estimates.

**Table 1.** Randomized trials reporting clinical outcomes of systematic AF screening.

Study	Number randomized	Screening intervention	Population	Primary outcome(s)
EARLY (2015)	928	Electrocardiogram every 6 months during a 2-year follow-up	Age ≥65 with risk factors, Spain	AF diagnosis
REHEARSE-AF (2017)	1 001	Hand-held ECG, BID for 1 year	Age 65 with risk factors, UK/Wales	AF diagnosis
LOOP (2021)	6 004	Implanted monitor	Age ≥70 with risk factors, Denmark	Stroke or systemic arterial embolism
SCREEN-AF (2021)	822	14-day ECG patch, twice	Age ≥75 with hypertension, Canada/Germany	AF diagnosis
STROKESTOP (2021)	28 768	Hand-held ECG, BID for 14 days	Age 75–76, Sweden	Combined endpoint of ischaemic or haemorrhagic stroke, systemic embolism, bleeding leading to hospitalisation, and all-cause death
GUARD-AF (2024)	11 905	14-day ECG patch	Age ≥70	All-cause stroke and bleeding
STROKESTOP II (2024)	28 712	Hand-held ECG, once if low NT-proBNP, otherwise GID for 14 days	Age 75–76, guided by NT-proBNP levels, Sweden	All-cause stroke and systemic embolism

**Figure 2.**
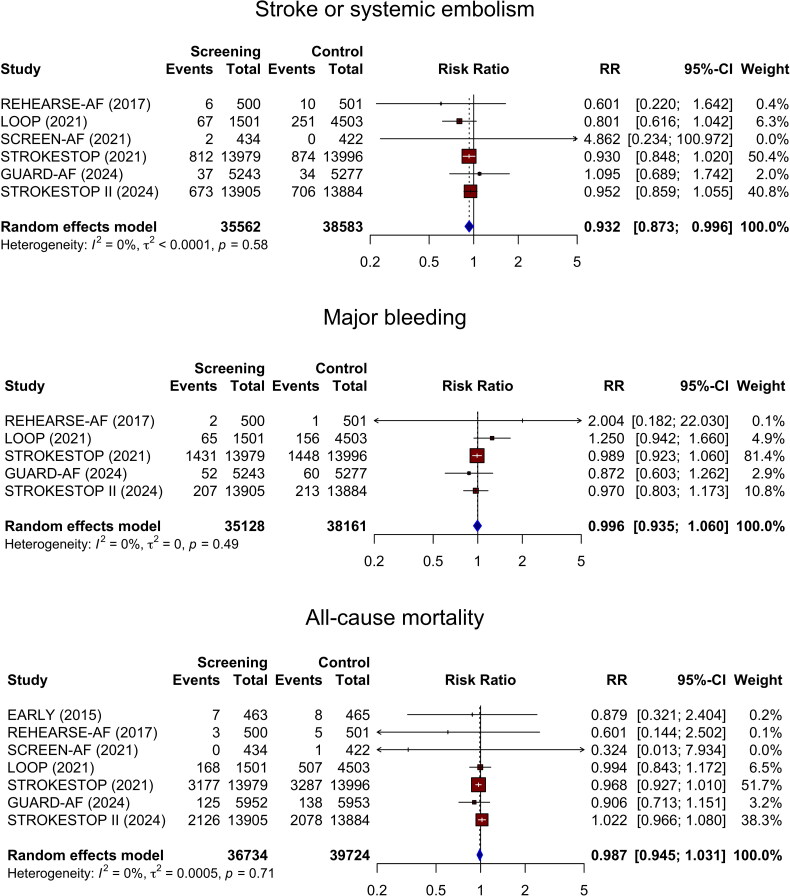
Forest Plot for the pairwise meta-analysis of stroke or systemic embolism, major bleeding, and all-cause mortality in screening group versus no screening.

Six studies reported data on stroke or systemic embolism, with none having individually statistically significant results. The pooled analysis showed that AF screening was associated with a reduction in stroke risk, with no heterogeneity across studies, although the confidence interval and p-value indicated only marginal statistical significance for this finding (Figure 2; RR 0.932, 95% CI 0.873–0.996, I^2^ = 0%, p-value 0.037). Assuming a 10% baseline stroke risk over ten years, the pooled relative effect translated to an absolute stroke risk reduction of 0.7% (95% CI: 0.0004% − 1.3%) and a number needed to screen of 148 (95% CI: 79 - 2501). The sensitivity analysis including only studies with clinical outcomes as primary endpoints, yielded similar results (Figure S1). However, if only studies that used non-invasive screening methods were considered (excluding the LOOP trial), the pooled risk estimate was not statistically significant (Figure S2; RR 0.942, 95% CI 0.880–1.008, I^2^ = 0%, p-value 0.083).

Five studies reported bleeding outcomes, all with neutral findings, and their pooled risk estimate for major bleeding with AF screening was also nonsignificant (Figure 2; RR 0.996, 95% CI 0.935–1.060, I^2^ = 0%, *p* = 0.876). The analysis limited to studies with clinical outcomes as primary endpoints showed similar results (Figure S1). Excluding the LOOP trial and focusing only on studies using non-invasive screening methods also yielded consistent findings (Figure S2).

Seven studies reported all-cause mortality, with none revealing statistically significant differences between screening and control groups. Correspondingly, the pooled results were nonsignificant (Figure 2; RR 0.987, 95% CI 0.945–1.031, I^2^ = 0%, *p* = 0.550). Results were consistent when considering only studies with clinical outcomes as primary endpoints and when including studies that used non-invasive methods (Figures S1 and S2).

## Discussion

This meta-analysis on the clinical outcomes of systematic electrocardiogram-based AF screening in individuals without known AF has three key findings: 1) AF screening is associated with a marginally statistically significant 7% relative reduction in the incidence of stroke and systemic embolism, 2) there is no signal of an increase harm in terms of major bleeding events, and 3) screening has no effect on all-cause mortality.

The increased implementation of oral anticoagulation for stroke prevention has been pivotal in improving the prognosis of patients with clinical AF and reducing the burden of stroke on both patients and healthcare systems over the past decades [15–17]. Additionally, there have been efforts to reduce stroke burden not only in patients with clinically detected AF but also in the large population of individuals with asymptomatic and undiagnosed forms of AF. However, these efforts have not achieved the same level of success in stroke prevention as those implemented for patients with clinically detected AF [18,19]. Studies investigating AF screening have generally shown neutral effects on clinical outcomes. Notably, only the STROKESTOP trial demonstrated a statistically significant reduction in its primary outcome—a composite of ischemic or hemorrhagic stroke, systemic embolism, hospitalization for bleeding, and all-cause mortality (hazard ratio 0.96; 95% CI: 0.92–1.00)—while the reduction in ischemic strokes did not reach statistical significance (hazard ratio 0.92; 95% CI: 0.83–1.01). Two meta-analyses on AF screening conducted some years ago exhibited discrepancies in their pooled estimates of clinical outcomes, reflecting differences in study selection criteria [6,7]. Furthermore, the two recently published large-scale AF screening trials, STROKESTOP II and GUARD-AF, failed to demonstrate a significant effect on stroke, with even their point estimates suggesting conflicting directions of possible effect [8,9]. The GUARD-AF trial was prematurely halted due to the COVID-19 pandemic, significantly reducing its power to detect any effect of screening. Thus, there has been considerable uncertainty about whether AF screening provides tangible clinical benefits.

The current meta-analysis provides an update on the evidence of the outcome effects of AF screening. The pooled risk estimates suggest that earlier detection of AF through systematic screening and subsequent anticoagulant therapy may indeed result in a 7% relative reduction in strokes. Despite considerable methodological differences between the trials, no statistical heterogeneity in treatment effects was observed across the studies. The pooled relative effect size translated to a number needed to screen of 148 to prevent one stroke over the next decade in a population of 75-year-olds. However, the wide confidence intervals reflect considerable uncertainty in this estimate, and the number needed to treat values depend on the real-life baseline stroke risk. Despite the seemingly modest relative effect, it is important to recognize that the absolute number of strokes prevented and the potential gain in quality-adjusted life years could be substantial in screened populations, given the high incidence of stroke in older adults and the severity of AF-related strokes.

Although accumulating evidence appears to suggest that AF screening may be effective in preventing strokes, the optimal screening methods and target populations have yet to be determined. More studies are needed to determine a threshold for clinically significant AF burden to screen for. Non-invasive repeated rhythm testing may have the advantage of more reliably detecting cases that represent a meaningfully high subclinical AF burden, in contrast to continuous invasive long-term monitoring, as in the LOOP trial, where the burden of AF in detected cases was low (median 0.13%) [20]. Of importance, in our analysis, the pooled risk estimates for stroke from studies employing solely non-invasive methodologies did not reach statistical significance, underscoring the need for further research in this area. Additionally, the marginal statistical significance and small pooled effect size render the current evidence susceptible to potential publication bias. While statistical evaluation of publication bias is challenging due to the limited number of studies, it is crucial that all trial data, even those with neutral outcomes, are published to ensure a comprehensive unbiased understanding of the effects of AF screening [14,21]. In this regard, it is important to note that the VITAL-AF screening trial, which enrolled over 30 000 participants, reported neutral results for its primary outcome of AF detection in early 2022. Data on the prespecified secondary outcomes, including stroke and major bleeding, have not been published, but given the neutral primary outcome, these are likely also neutral [22–24].

Moreover, the pooled results are largely driven by the two large STROKESTOP studies, wherein the control group received no information about the study. As a result, the Hawthorne effect—where participants modify their behavior simply due to being observed—affected only the intervention group. This secondary effect of screening intervention may partially explain the observed stroke reduction in the STROKESTOP studies, especially given that anticoagulation use was similar between the control and screening groups in both studies [9,12]. Therefore, other factors beyond AF detection and subsequent anticoagulation may contribute to the improved outcomes seen with screening. For example, the screening invitation may encourage participants to seek medical care for other stroke risk factors, potentially leading to better control of hypertension, dyslipidemia, and diabetes. Before large-scale AF screening protocols are implemented in practice, further investigation is needed to confirm that the benefits suggested by the pooled estimates are truly attributable to AF screening *per se*.

The incidence of AF is on the rise, driven in part by greater longevity and improved survival rates from other cardiac conditions, but also by increased awareness and advancements in diagnostics [5,10,12]. Moreover, there has been an influx of novel consumer-based devices for heart rhythm monitoring in recent years. These advancements may have led to increased detection of subclinical AF, even without specific screening interventions. Moreover, related to improved diagnostics, as well as improved treatment of comorbidities and stroke risk factors, the initial non-anticoagulated stroke risk of patients with newly diagnosed AF has decreased over time [25]. These temporal trends may have diminished the potential yield of systematic AF screening, limiting its impact on stroke outcomes in contemporary settings. Another important consideration regarding the public health benefits of screening is that individuals who choose to participate tend to be healthier and of higher socioeconomic status than those who do not [9,12,26]. Unfortunately, those most reluctant to participate are often the ones who would be most likely to benefit from screening. This aspect has complicated clinical trials in demonstrating the potential stroke reduction benefits of AF screening and is likely to similarly limit the benefits in real-world settings.

The pooled estimates for major bleeding and all-cause mortality do not signal significant harms associated with AF screening. Given the large number of participants and the proximity of the pooled estimates to unity, it is unlikely that screening has a clinically meaningful impact on major bleeding or all-cause mortality. The somewhat unexpected absence of an increased bleeding risk may reflect the comparable use of OACs between the screening and control groups in the large STROKESTOP trials [9,12]. However, data from the LOOP trial indicated that increased incidental detection of asymptomatic bradyarrhythmias through screening may lead to unnecessary implantation of pacemakers, without any observed differences in syncope or sudden death between screened and unscreened individuals [27,28]. Additionally, screening can yield false-positive findings or results that necessitate further confirmatory testing, potentially leading to increased patient anxiety, healthcare costs, and again risk of new incidental findings [29]. Furthermore, optimal management of infrequent short AF episodes remains uncertain, and in some cases, the use of oral anticoagulation may result in net harm [19]. It is possible that not all downstream adverse consequences resulting from screening have been captured in the trials, which complicates the estimation of these potential harms in the decision-making process regarding screening interventions. These data would also be needed to determine cost-effectiveness of screening, a key element of policy-making. Nevertheless, although there has been lack of data on all adverse effects and insufficient robust evidence supporting the initial assumption that AF screening effectively prevents strokes, some previous mathematical decision modeling studies have suggested that screening strategies could be cost-effective in older populations [30,31].

Limitations of the current study need to be considered. First, due to the limited number of studies, publication bias cannot be statistically assessed, and if present, it could affect particularly the pooled estimates of stroke prevention. Second, this study utilized a study-level meta-analysis rather than a patient-level approach, limiting our ability to perform granular analyses to identify subgroups of patients who may benefit most from systematic screening. Third, we combined results from studies that used different screening tools and randomized patients at various stages of the study protocol—such as the STROKESTOP trials, which randomized at the population level for screening invitations versus no invitation, while others randomized after trial participation consent for screening versus no screening. Lastly, only English-language studies were included, because large AF screening trials are unlikely to be published in other languages.

In conclusion, this meta-analysis provides an updated assessment of the clinical outcomes of systematic AF screening, showing that screening is associated with a 7% reduction in stroke incidence and has no significant impact on major bleeding or all-cause mortality. However, more research is needed to determine optimal screening methods and whether factors beyond AF detection and subsequent anticoagulation contribute to the observed reduction in stroke risk.

## Supplementary Material

Supplemental Material

## Data Availability

We have deposited the codes used in these analyses in the Zenodo repository, where they are openly available under DOI: 10.5281/zenodo.13984037. The codes are accessible *via* its DOI through the Digital Object Identifier Foundation’s search portal at https://www.doi.org, or *via* this direct link: https://zenodo.org/records/13984038. All data and codes data will be shared upon request made to the corresponding author of the manuscript.
