# Supplementary material for: Clinical outcomes of atrial fibrillation screening: a meta-analysis of randomized controlled trials
Source: Ann Med. 2025 Jan 25;57(1):2457522. doi: 10.1080/07853890.2025.2457522 (PMC12161479; doi:10.1080/07853890.2025.2457522)
Supplement: Supplemental Material [file IANN_A_2457522_SM1113.zip › Suppl/SupplementaryMaterialRevision.docx]

**Supplementary Material**

**Supplementary Figure 1.** Forest plot for the pairwise meta-analysis of stroke or systemic embolism, major bleeding, and all-cause mortality in screening group versus no screening including only studies clinical endpoints as the primary outcome

**Supplementary Figure 2.** Forest plot for the pairwise meta-analysis of stroke or systemic embolism, major bleeding, and all-cause mortality in screening group versus no screening including only studies using non-invasive screening methods (excluding the LOOP trial)

**Supplementary Figure 1.** Forest plot for the pairwise meta-analysis of stroke or systemic embolism, major bleeding, and all-cause mortality in screening group versus no screening including only studies with clinical endpoints as the primary outcome

**Supplementary Figure 2.** Forest plot for the pairwise meta-analysis of stroke or systemic embolism, major bleeding, and all-cause mortality in screening group versus no screening including only studies using non-invasive screening methods (excluding the LOOP trial)
